# Life-Threatening Intraventricular Rupture of Brain Abscess in a Patient With Undiagnosed Hereditary Hemorrhagic Telangiectasia

**DOI:** 10.7759/cureus.8732

**Published:** 2020-06-21

**Authors:** Christopher M Nguyen, Jessica Stauber, Michelle Baliss, David Reynoso

**Affiliations:** 1 Internal Medicine, University of Texas Medical Branch at Galveston, Galveston, USA; 2 Medicine, University of Texas Medical Branch at Galveston, Galveston, USA; 3 Infectious Disease, University of Texas Medical Branch at Galveston, Galveston, USA

**Keywords:** ventriculitis, septic emboli, hereditary hemorrhagic telangiectasia, osler-weber-rendu, pulmonary arteriovenous malformation, brain abscess, arteriovenous malformation, intraventricular rupture of brain abscess

## Abstract

Hereditary hemorrhagic telangiectasia (HHT) is an autosomal dominant genetic condition associated with mucocutaneous and visceral arteriovenous malformations (AVMs), including pulmonary AVMs, which predispose patients to systemic paradoxical emboli that can lead to brain abscesses. Intraventricular rupture of brain abscess (IVROBA) is a feared complication with a high mortality rate. Here, we present a case with brain abscesses complicated by IVROBA and ventriculitis as the initial presentation of HHT in an undiagnosed patient. We also discuss the diagnostic and therapeutic approach that resulted in this patient’s clinical improvement.

## Introduction

Hereditary hemorrhagic telangiectasia (HHT), also known as Osler-Weber-Rendu syndrome, is a rare disorder characterized by vascular wall defects causing mucocutaneous and visceral arteriovenous shunts that can occur throughout the body [1]. The Curacao criteria for HHT diagnosis require the presence of three or more of the following: recurrent epistaxis, characteristic mucocutaneous telangiectasias, visceral involvement, and first-degree relatives with HHT. Small vessel telangiectasias occur more frequently and lead to recurrent mucocutaneous bleeding such as epistaxis, which affects 95% of patients with HHT [1]. More serious and often catastrophic implications arise when larger vessel arteriovenous malformations (AVMs) occur in the internal organ vasculature, such as pulmonary, gastrointestinal, and cerebral circulations. Pulmonary AVMs (PAVMs) affect 15%-45% of patients with HHT and result in paradoxical systemic emboli through right-to-left shunting, which bypasses the filtering ability of the pulmonary capillary bed and leads to an increased propensity for embolic strokes and brain abscesses (BAs) by multiple mechanisms that are not completely understood [2,3]. One potentially devastating complication is the risk of intraventricular rupture of brain abscesses (IVROBA), which if left untreated, carries a high mortality rate [4]. We report a case of a patient with undiagnosed HHT with IVROBA and ventriculitis as the presenting manifestations of his condition.

## Case presentation

**Figure 1:**
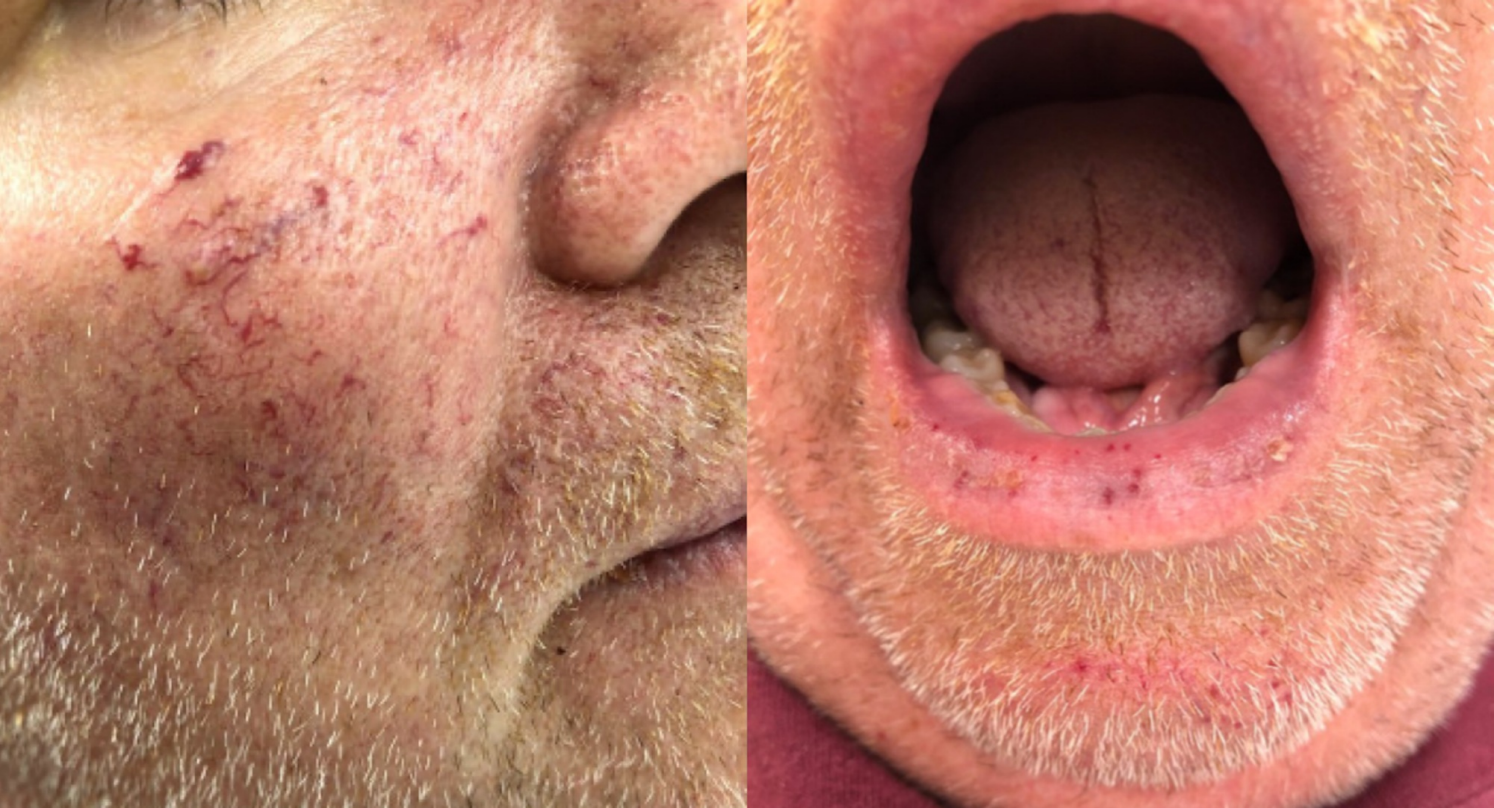
Mucocutaneous telangiectasias Multiple mucocutaneous telangiectasias.

A 60-year-old male with a history of recurrent epistaxis since childhood presented with four weeks of intermittent fevers, fatigue, new-onset word-finding difficulties, slurred speech, headaches, blurred vision, and nausea. Upon presentation, he was febrile and tachycardic. Physical exam was notable for delayed speech, impaired concentration, multiple mucocutaneous telangiectasias (Figure 1), and left-sided weakness. Further investigation revealed a history of recurrent gastrointestinal bleeding in his mother and a sister with recurrent epistaxis since childhood. Labs were significant for leukocytosis (white blood cell count [WBC] 16 × 10^3^/µL) with a neutrophilic predominance.

**Figure 2:**
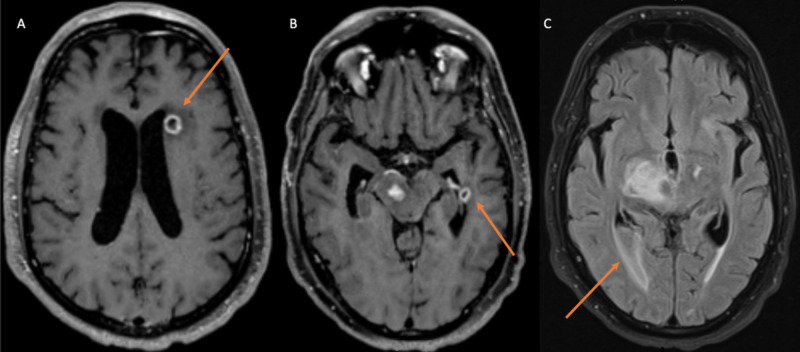
Intraventricular rupture of brain abscess (A) The arrow indicating brain abscess adjacent to the left ventricle. (B) The arrow indicating brain abscess abutting the left ventricle. (C) The arrow indicating intraventricular rupture of abscess into the posterior horn of the right lateral ventricle.

**Figure 3:**
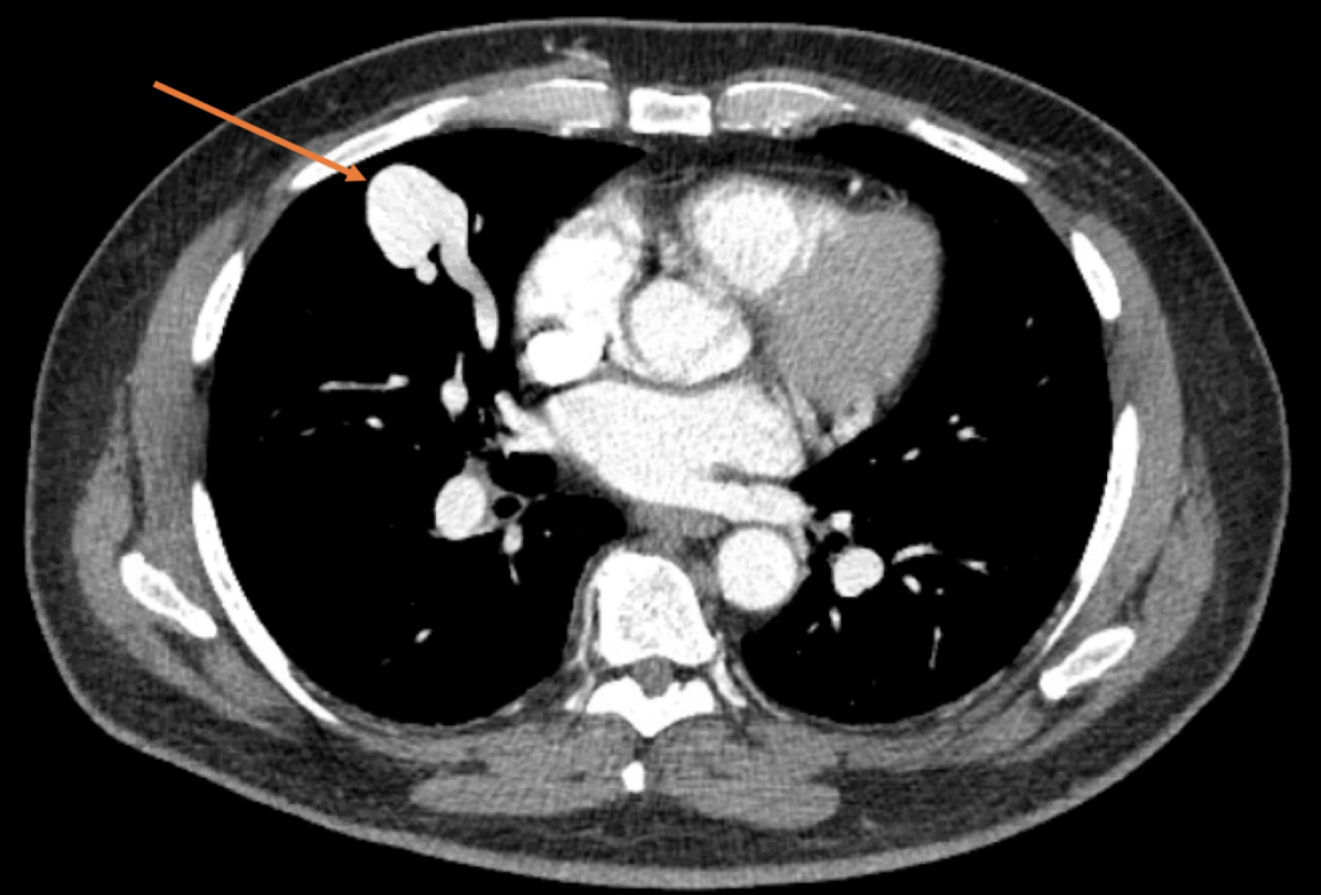
Pulmonary arteriovenous malformation Pulmonary arteriovenous malformation measuring 2.8 cm in diameter.

CT of the head showed a frontal lobe hypoattenuating lesion. MRI revealed multiple supratentorial BAs with rupture into the right lateral ventricle and evidence of ventriculitis (Figures 2A-2C). CT of the thorax showed two large PAVMs measuring 2.8 cm (Figure 3) and numerous hepatic AVMs. At this point, the diagnosis of HHT was confirmed based on the Curacao diagnostic criteria [1,5]. Lumbar puncture (LP) yielded grossly purulent cerebrospinal fluid with WBC of 7,750/µL and 93% segmented neutrophils. Multiple cultures of blood, urine, and cerebrospinal fluid resulted negative throughout his hospitalization. He was started on ceftriaxone, metronidazole, and vancomycin. However, he demonstrated minimal clinical improvement within the first week of therapy, and the spectrum of antibacterial coverage was escalated to meropenem. He underwent transcatheter embolization of the PAVMs, resulting in significant improvement in mentation. CT of the head was repeated and demonstrated resolution of the ventriculitis and interval improvement of the abscesses. LP was repeated 15 days later and showed resolution of neutrophilic pleocytosis. His mentation continued to improve, and he remained hemodynamically stable. He was discharged with plans to complete six weeks of meropenem in the outpatient setting. 

## Discussion

HHT is rare, with a prevalence ranging between 1:5,000 and 1:8,000; however, it is likely underreported due to lack of public awareness and the generally benign presentation [1]. The most common manifestations of HHT are epistaxis (95%), PAVMs (50%), hepatic AVMs (30%), gastrointestinal bleeding (20%), and central nervous system AVMs (10%). Mutations in genes affecting the function of vascular endothelial cells during angiogenesis are responsible for the dilatations that lead to AVM formation [1].

The overwhelming majority of PAVM cases are associated with HHT, as 15%-45% of patients with HHT have PAVMs and 80%-90% of patients with PAVMs are eventually diagnosed with HHT [3,6]. The genetic mutations in HHT cause abnormally dilated pulmonary vessels which eventually lead to AVMs that allow for right-to-left shunting [2]. This predisposes HHT patients to paradoxical emboli and the potential for ischemic strokes and BAs [2,6]. The propensity for neurological manifestations increases with larger sized feeding arteries (>3 mm) and with increasing number of PAVMs [7]. Several proposed mechanisms of BA formation secondary to HHT exist; however, the majority implicate the pulmonary capillaries as either a crucial mechanical filter of septic emboli or being a major contributor to the body’s adaptive immune system attributable to the presence of cytokines, macrophages, and other immunoregulators in close proximity to the capillaries [8,9]. It could also be hypothesized that gastrointestinal and hepatic AVMs would allow a greater baseline translocation of gut bacteria into the portal system through the inferior vena cava, ultimately entering the right heart circulation, and contributing to BA formation in the presence of PAVMs. Although HHT is an uncommon clinical entity, PAVMs can also be acquired by more common conditions, such as trauma, surgeries, certain parasitic or bacterial infections, hepatopulmonary syndrome, mitral stenosis, and cancer [6]. It is therefore crucial for physicians to recognize the potentially devastating neurological consequences of PAVMs and the considerable reduction in morbidity and mortality with timely recommended treatments such as embolotherapy or surgery for symptomatic or large PAVMs. Preprocedural prophylactic antibiotics should also be offered to patients with PAVMs to reduce the risk of infectious seeding [10].

BAs represent a serious complication of HHT and generally present with systemic signs of infection, focal neurological deficits, or signs of increased intracranial pressure. Although BAs present similarly in HHT as they do in the general population, the risk of BAs in patients with HHT is evidently 1,000 times greater than the general population [3,11]. Anaerobic and facultative anaerobic bacteria are frequently implicated in both populations, with Streptococcus species being the most common in both [3,11,12]. However, compared to BAs in the general population, Staphylococcus is rarely isolated from BAs in HHT in which BAs are more likely to be polymicrobial [3,12,13]. Approximately 20%-30% of BAs in HHT patients will have sterile cultures for unclear reasons, as was the case in our patient [3,11]. One possible explanation for the sterile cultures could be the use of antibiotics prior to the LP. IVROBA is a rare and often catastrophic complication of BA with a mortality rate of 90% due to late presentation with abrupt deterioration and is seldom documented in the setting of HHT [4,12]. Some evidence has shown, however, that early detection by CT scan of meningeal irritation and ventricular wall enhancement adjacent to the abscess, which precedes IVROBA, could prompt early aggressive treatment and reduce the mortality rate to approximately 40% [4]. Suggested treatments include both intravenous and intrathecal antibiotics with image-guided aspiration prior to IVROBA [4]. A retrospective study done on the rare cases of IVROBA survival showed that more aggressive neurosurgical intervention may be necessary after rupture, with external ventricular drains being the most preferred [14]. Despite presenting after IVROBA, our patient survived and stabilized with aggressive systemic antibiotic therapy alone before he requested to be discharged. On follow-up three weeks after discharge, CT of the head showed further improvement of his abscesses.

## Conclusions

Our case illustrates that an IVROBA causing ventriculitis could be the initial presentation of a patient with undiagnosed HHT. This highlights the need for early screening for PAVMs in patients with a known history of HHT that could predispose them to BAs. This also highlights the need for early screening of BAs when neurological deficits are present to prevent their rupture and reduce mortality as well as long-term neurologic consequences. This case also serves to raise clinician awareness of other common medical conditions that can lead to acquired PAVM formation. Thus, it is important to offer these patients preprocedural prophylactic antibiotics and embolotherapy when clinically appropriate to reduce morbidity and mortality.

## References

[1] Kuhnel T, Wirsching K, Wohlgemuth W, Chavan A, Evert K, Vielsmeier V (2018). Hereditary hemorrhagic telangiectasia. Otolaryngol Clin North Am.

[2] Etievant J, Si-Mohamed S, Vinurel N (2018). Pulmonary arteriovenous malformations in hereditary haemorrhagic telangiectasia: correlations between computed tomography findings and cerebral complications. Eur Radiol.

[3] Mathis S, Dupuis-Girod S, Plauchu H (2012). Cerebral abscesses in hereditary haemorrhagic telangiectasia: a clinical and microbiological evaluation. Clin Neurol Neurosurg.

[4] Takeshita M, Kawamata T, Izawa M, Hori T (2001). Prodromal signs and clinical factors influencing outcome in patients with intraventricular rupture of purulent brain abscess. Neurosurgery.

[5] Shovlin CL, Guttmacher AE, Buscarini E (2000). Diagnostic criteria for hereditary hemorrhagic telangiectasia (Rendu-Osler-Weber syndrome). Am J Med Genet.

[6] Saboo SS, Chamarthy M, Bhalla S (2018). Pulmonary arteriovenous malformations: diagnosis. Cardiovasc Diagn Ther.

[7] Moussouttas M, Fayad P, Rosenblatt M (2000). Pulmonary arteriovenous malformations: cerebral ischemia and neurologic manifestations. Neurology.

[8] Shovlin C, Bamford K, Sabba C (2019). Prevention of serious infections in hereditary hemorrhagic telangiectasia: roles for prophylactic antibiotics, the pulmonary capillaries-but not vaccination. Haematologica.

[9] Iwasaki A, Foxman EF, Molony RD (2017). Early local immune defenses in the respiratory tract. Nat Rev Immunol.

[10] Gossage JR, Kanj G (1998). Pulmonary arteriovenous malformations. A state of the art review. Am J Respir Crit Care Med.

[11] Larsen L, Marker CR, Kjeldsen AD, Poulsen FR (2017). Prevalence of hereditary hemorrhagic telangiectasia in patients operated for cerebral abscess: a retrospective cohort analysis. Eur J Clin Microbiol Infect Dis.

[12] Tseng JH, Tseng MY (2006). Brain abscess in 142 patients: factors influencing outcome and mortality. Surg Neurol.

[13] Lu CH, Chang WN, Lin YC (2002). Bacterial brain abscess: microbiological features, epidemiological trends and therapeutic outcomes. QJM.

[14] Omar AT II, Khu KJO (2020). Successful management of intraventricular rupture of pyogenic brain abscess (IVROBA): systematic review and illustrative case. J Clin Neurosci.

